# Nature‐Inspired Histidine and Guanidine Modification of Magnetic Nanoparticles for Urinary DNA Liquid Biopsy

**DOI:** 10.1002/smtd.71034

**Published:** 2026-09-18

**Authors:** Dina Haddad, Ishtiaq Ahmed, Manthos Pitoulias, Richard Mair, Ljiljana Fruk

**Affiliations:** ^1^ Chemical Engineering and Biotechnology (CEB) University of Cambridge Cambridge UK; ^2^ Cancer Research UK Cambridge Institute (CRUK) University of Cambridge Cambridge UK

**Keywords:** cell‐free DNA, DNA extraction, magnetic nanoparticles, urine‐biopsy

## Abstract

Urinary cell‐free DNA (cfDNA) is a promising substrate for non‐invasive liquid biopsy analysis and biomarker detection. However, the lack of standardized pre‐analytical workflows and the intrinsic challenges associated with isolating highly dilute and fragmented cfDNA limit its broader research and clinical applications. Here, we present a simplified, reliable and cost‐effective extraction platform based on iron oxide nanoparticles (IONPs) synthesized via coprecipitation. By leveraging nature‐inspired surface functionalization, these magnetic nanoparticles enable the rapid and direct isolation of cfDNA from fresh urine while supporting scale‐up of the processing volumes. The proposed platform achieves a 2‐fold increase in cfDNA yield compared to conventional commercial extraction kits and demonstrates enhanced recovery of short DNA fragments, with a predominant size distribution between 80–150 bp. This improved capture efficiency facilitates a detection sensitivity down to 10^−3^ ng/mL using qPCR, likely limited by assay sensitivity rather than DNA extraction performance. Moreover, the simplified extraction and elution steps eliminate the need for specialized laboratory instrumentation. Collectively, this approach provides a foundation for accessible, potentially at‐home cfDNA extraction and advances the development of truly non‐invasive diagnostic strategies for longitudinal disease monitoring and management.

## Graphical abstract

Nature‐inspired bi‐functionalized magnetic nanoparticles are engineered for the rapid, non‐invasive extraction of cell‐free DNA from unprocessed urine for liquid biopsy. These cationic particles leverage electrostatic interactions to outperform conventional kits, enhancing the recovery of short DNA fragments while maintaining DNA integrity. By eliminating centrifugation, this platform enables accessible, home‐based liquid biopsy for sensitive longitudinal disease monitoring.

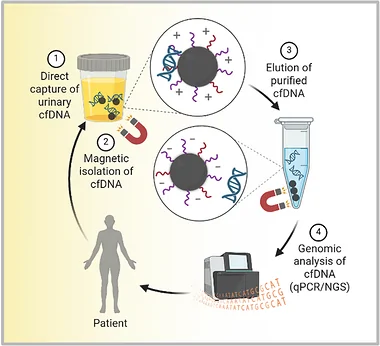

## 1 Introduction

Tissue biopsy remains the diagnostic gold standard for cancer; however, it is inherently invasive and associated with significant morbidity and, in some cases mortality, depending upon the biopsy location. Furthermore, tissue biopsy provides only a spatiotemporal snapshot of the tumor and fails to capture the full extent of tumor heterogeneity [1, 2, 3]. Multiple biopsies are rarely feasible due to procedural risk, cost, and patient burden, thereby limiting its utility for longitudinal monitoring of treatment response. In contrast, liquid biopsy approaches, most commonly based on the analysis of cell‐free DNA (cfDNA), provide a minimally invasive alternative for dynamic tumor assessment using accessible biofluids including blood, saliva, and urine [4]. Beyond oncology, liquid biopsies have demonstrated clinical utility in non‐neoplastic contexts, including non‐invasive prenatal testing and the detection of transplant organ rejection, thereby expanding the scope of these analytical strategies [5, 6].

cfDNA is released from cells into biofluids primarily through apoptosis, necrosis, and active secretion, and retains genomic and epigenomic features reflective of its cell of origin [7]. The modal fragment size of cfDNA is typically ∼166 bp [8, 9], corresponding to DNA wrapped around a nucleosome with associated linker DNA. In contrast, tumor‐derived cfDNA (ctDNA) often exhibits a shorter modal fragment size (∼134–144 bp) [10, 11], likely arising from altered chromatin structure and increased atypical endonuclease activity in cancer cells. Fragmentation is further pronounced in urine, where ctDNA is typically observed in the 80–120 bp range, potentially due to elevated nuclease activity within the urinary tract [11, 12].

Crucially, urine can capture ctDNA from both urological and non‐urological malignancies. While urological cancers shed longer fragments (>1000 bp) directly into the bladder, short fragments (40–250 bp) from systemic diseases can successfully cross the glomerular basement membrane and be filtered into the urine (Figure 1A) [3, 13]. Given that the half‐life of circulating cfDNA is exceedingly short (15 min to 2.5 h) [14, 15, 16], liquid biopsies offer a real‐time snapshot of disease burden.

**FIGURE 1:**
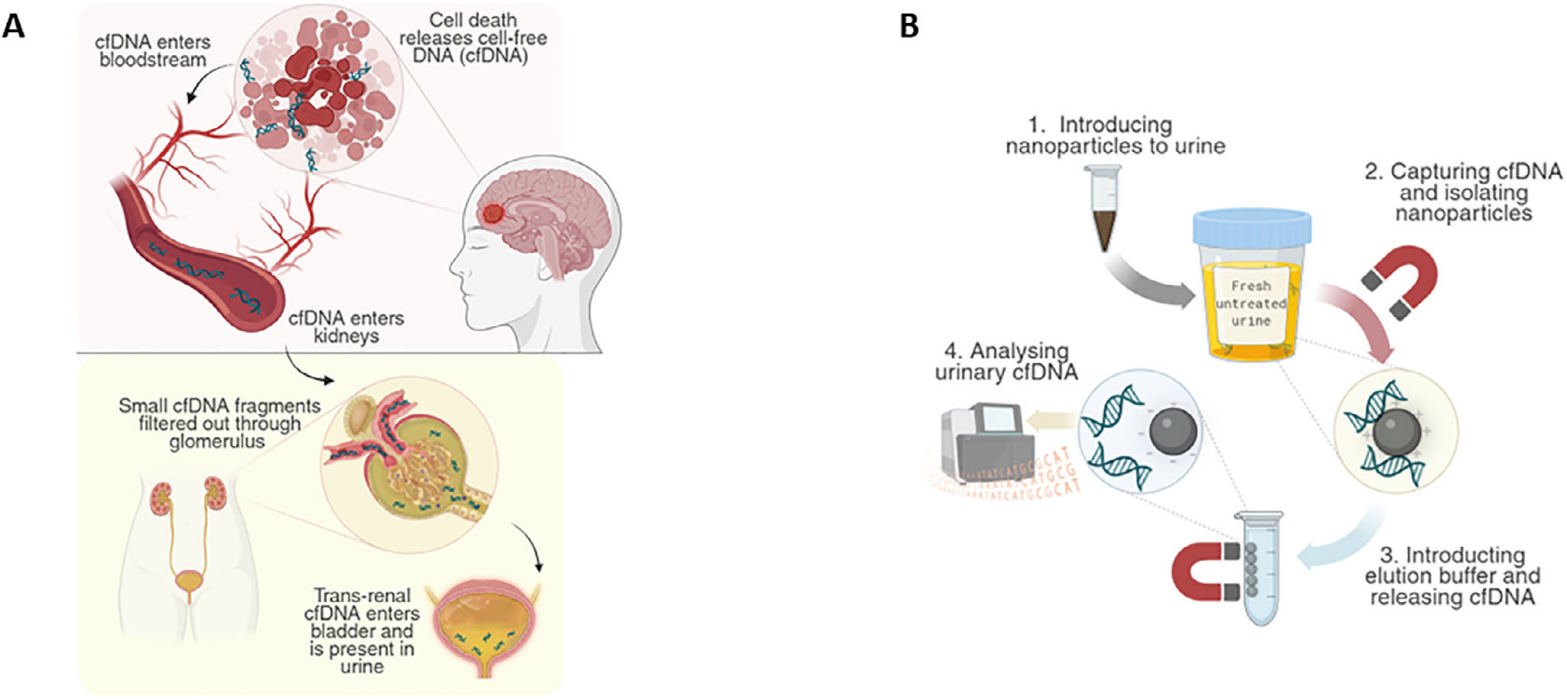
Origin of urinary cfDNA and the proposed extraction platform. (A) Biological mechanism of transrenal cfDNA shedding, demonstrating how short cfDNA fragments from systemic tumors cross the glomerular basement membrane and are filtered into the urine. (B) Simplified schematic representing the core concepts and protocol for the extraction of cfDNA from urine using magnetic nanoparticles.

While ctDNA is a powerful tool for non‐invasive diagnosis, therapy monitoring, clinical trial assessment, and prognosis [17], its clinical translation remains hindered by its low abundance in biofluids (1–200 ng/mL) [18] and severe pre‐analytical handling challenges. Blood‐derived liquid biopsies are restricted by limited sampling volumes and the inconvenience of repeated draws that require venesection. In contrast, urine provides a completely non‐invasive matrix that can be collected in large volumes and at high frequencies [18]. However, delays in processing cause leukocyte lysis, genomic DNA contamination, and rapid cfDNA degradation [19], exacerbated by temperature fluctuations and freeze‐thaw cycles. Therefore, technologies enabling immediate cfDNA capture from untreated biofluids at the point of collection are a useful means of preserving useful analyte purity and improving assay reliability.

Current extraction methodologies remain unsuited for point‐of‐care or at‐home use. Commercial extraction kits (e.g., Zymo, MagMax, QIAamp) [20, 21] are labor‐intensive, requiring multiple centrifugation steps and rely on silica‐ or bead‐based adsorption mechanisms that necessitate chaotropic salts [20, 22, 23, 24]. Conventional chemical approaches, such as phenol‐chloroform extraction or isopropanol precipitation [25], are even more hazardous and thus unsuitable for routine clinical use (Table S1).

Nanomaterials present an attractive alternative due to their high surface‐to‐volume ratio and tunable surface chemistry, making them ideal for capturing low‐abundance analytes from large volumes. Their surfaces can be functionalized to interact with DNA via electrostatic forces [26], hydrogen bonding [27], or π–π stacking [28, 29], although urine matrix effects such as high ionic strength and urea content can significantly impair binding efficiency [30]. Recent advances include zinc oxide nanowires for microfluidic urinary cfDNA capture [27] and hydroxyl‐functionalized magnetic nanoparticles for plasma extraction [31]. Despite these developments, a critical gap remains: most platforms are confined to microliter‐scale processing, resulting in insufficient nucleic acid yields, or they require urine pre‐processing steps that limit their applicability in decentralized, out‐of‐laboratory setting.

To address these limitations, we present a simple, scalable method for the direct extraction of low concentration cfDNA from untreated urine using cationic magnetic nanoparticles (Figure 1B). This system enables rapid DNA capture, directly from unprocessed urine, followed by efficient separation via an external magnetic field, thereby eliminating the need for centrifugation. Crucially, the approach is readily scalable to process volumes exceeding 100 mL without compromising performance. By integrating these nanoparticles into a simple, home‐compatible format, this platform allows for immediate cfDNA isolation at the point of collection, preserving molecular fidelity and enabling more reliable downstream analysis. This capability represents a meaningful step towards decentralized, non‐invasive cancer monitoring.

## 2 Results and Discussion

### 2.1 IONP Synthesis and Surface Modification

Iron oxide nanoparticles (IONPs) were synthesized using a modified co‐precipitation method [32, 33]. While co‐precipitation is simple and environmentally friendly, it is often limited by size heterogeneity and poor reproducibility due to rapid nucleation. Given that both nanoparticle size and magnetic saturation are highly sensitive to pH, the synthesis was adapted into a fed‐batch reaction to achieve improved control over pH and mixing dynamics (Figure S1). Iron precursors were continuously introduced at a constant rate into a reactor maintained at pH 12 yielding nanoparticles with a diameter of 7.6 nm (Figure 2A). However, bare IONPs have an isoelectric point (*pI*) between 7.0 and 7.5, resulting in diminished electrostatic stability and a strong tendency to aggregate at physiological pH (Figure S3). Furthermore, unmodified IONPs are prone to oxidation, potentially forming a magnetite/maghemite core/shell structure that reduces magnetic saturation in aqueous suspensions [34, 35].

**FIGURE 2:**
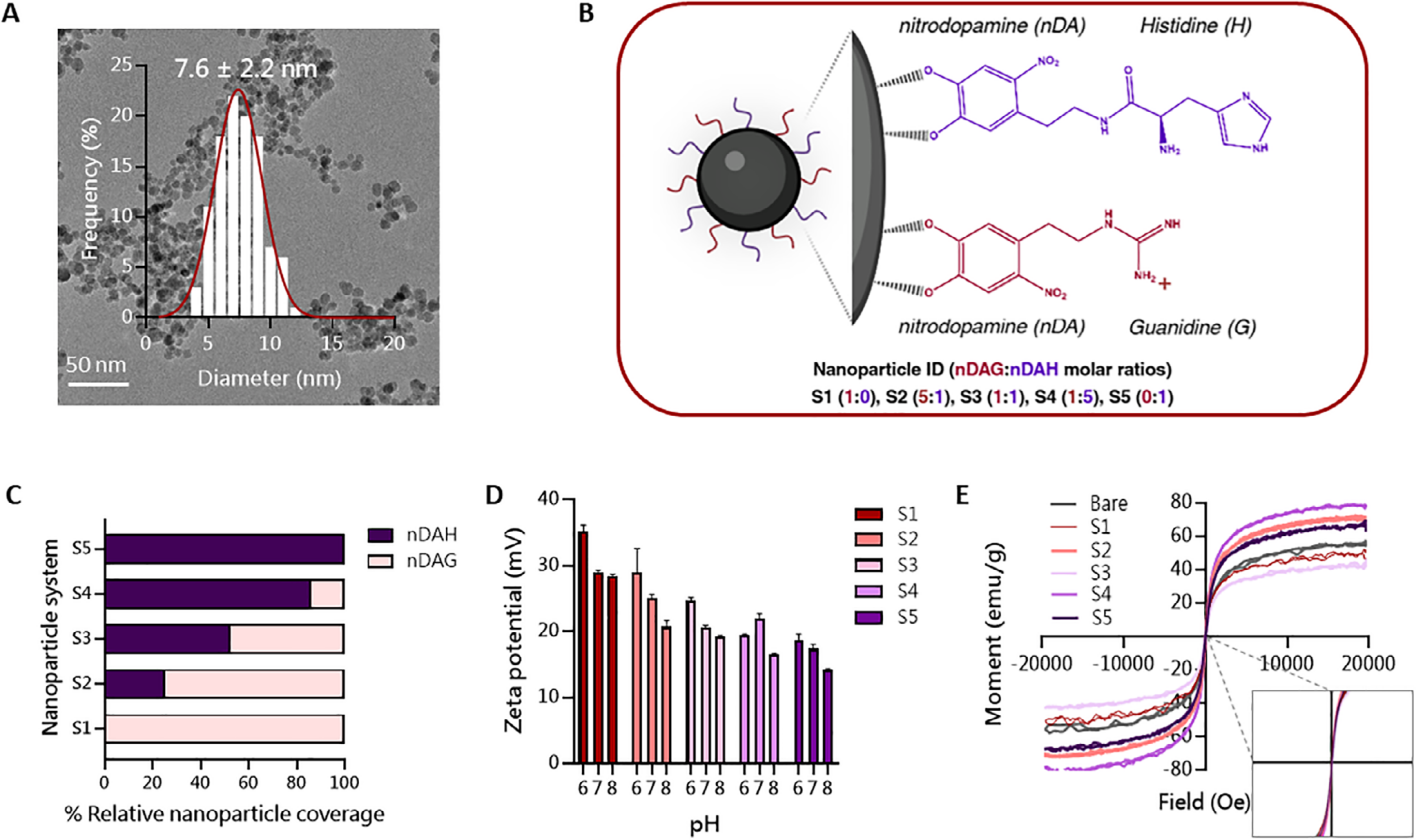
(A) Transmission electron microscopy (TEM) image of bare IONPs and size distribution histogram. (B) Schematic representation of nitrodopamine‐guanidine (nDAG) and nitrodopamine‐histidine (nDAH) chelation onto the surface of IONPs at different ratios of S1‐S5 formulations. (C) The relative composition of the surface coating of the IONPs calculated based on HPLC measurements of each linker. (D) Zeta potential of aqueous suspensions of S1‐S5 IONPs at urine pH range of 6–8. (E) Vibrating sample magnetometer (VSM) measurements indicating magnetic saturation of IONPs under alternating magnetic field and superparamagnetic properties.

To simultaneously prevent oxidative degradation, enhance colloidal stability, and enable the targeted capture of short double‐stranded cfDNA from urine, the IONPs were functionalized with nDAG and nDAH linkers at molar ratios of 1:0, 5:1, 1:1, 1:5, and 0:1, yielding systems S1 through S5, respectively (Figure 2B). This surface modification strategy was directly inspired by natural DNA‐histone interactions. The guanidine moiety (analogous to arginine, with a high p*K_a_*) facilitates strong electrostatic adsorption of the negatively charged dsDNA backbone, while the imidazole group (analogous to histidine, with a lower p*K_a_*) enables π–π stacking and allows efficient, non‐destructive DNA release via mild pH modulation.

Although direct detection of the linkers on the IONPs surface was not performed, high‐performance liquid chromatography (HPLC) analysis of the filtrate strongly suggested that the targeted linker compositions were successfully achieved (Figure 2C). This dual functionalization strategy resulted in a surface isoelectric point (*pI*) exceeding 8, and zeta potential measurements confirmed that all S1‐S5 formulations maintained a net positive charge across pH 6–8, ranging from strongly cationic (∼+30 mV for S1) to moderately cationic (∼+10 mV for S5) (Figure 2D). Consequently, these modified IONPs, establish robust electrostatic interactions for DNA binding while maintaining functionality in the high‐ionic‐strength environment of urine, where steric stabilization helps offset charge screening effects.

The magnetic saturation (*M_s_*) of the 7 nm particles, measured using a VSM over a field range of −20 to +20 kOe, was approximately 60 emu/g for IONPs dispersed in water (Figure 2E), consistent with reported values (45–70 emu/g) [36, 37] for magnetite IONPs of comparable size. The particles exhibited superparamagnetic behavior, with negligible remanence at 0 Oe, enabling rapid redispersion following magnetic separation. This property is advantageous for DNA desorption as it minimizes irreversible particle agglomeration and promotes uniform interaction with the elution buffer. While *M_s_* typically decreases for particles under 10 nm due to increased surface disorder [38], bare 13 nm IONPs synthesized via a microemulsion method exhibited a comparable *M_s_* of 60 emu/g (Figure S4). Therefore, the smaller particles (7.6 nm) were selected, as they provide a larger surface area while also benefiting from simpler and more cost‐effective synthesis (Figure S2).

Upon lyophilization, S1‐S5 IONPs displayed *Ms* ranging from 50 to 78 emu/g. This variation is attributed to differences in aggregation behavior during freeze‐drying; highly hydrophilic and colloidally stable particles (such as S1) likely remain more dispersed, resulting in lower apparent *M_s_*, whereas relatively hydrophobic systems (such as S5) are more prone to aggregation, yielding higher measured values. Overall, surface functionalization proved essential not only for enabling efficient DNA capture but also for preserving favorable magnetic properties.

### 2.2 Nanoparticle Binding to Double‐Stranded DNA in Synthetic Urine

To evaluate double‐stranded DNA (dsDNA) binding and elution, the nanoparticle systems (S1‐S5) were assessed using a synthetic TAMRA‐labeled 90 bp dsDNA in a synthetic urine matrix. The protocol was optimized by varying nanoparticle addition (2–100 µg) to ensure that maximum binding capacity was reached. Binding efficiency was quantified following a 5‐min incubation of IONPs and DNA at room temperature, after which the nanoparticles were magnetically separated. The concentration of unbound DNA remaining in the supernatant was then measured to determine binding capacity and refine nanoparticle dosing (Figure 3A).

**FIGURE 3:**
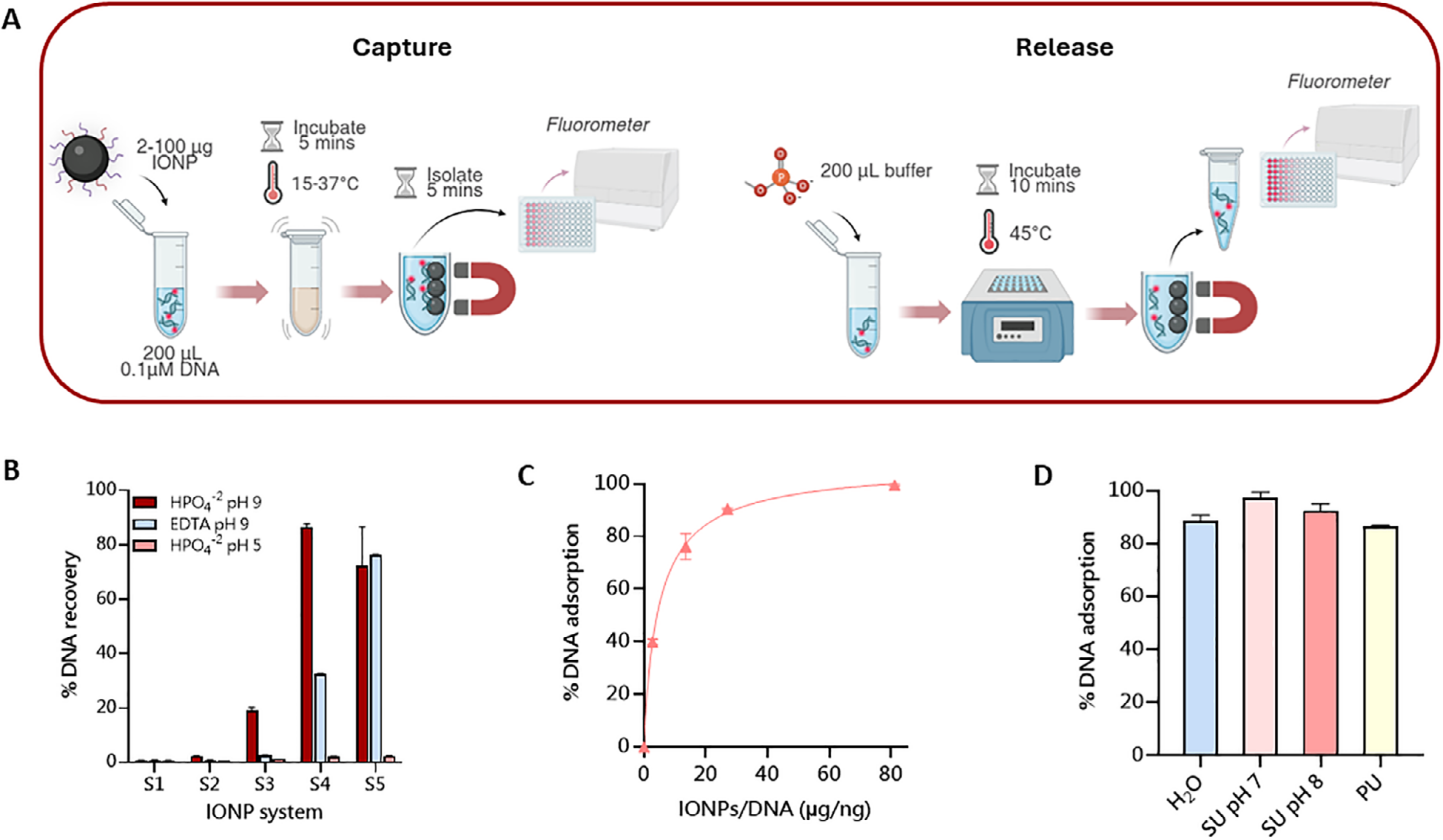
(A) Schematic representation of the experimental pipeline used for the capture and release of synthetic TAMRA‐labeled 90 bp DNA in synthetic urine. (B) Recovery of 90 bp DNA from S1‐S5 IONPs with different elution buffers. (C) DNA adsorption saturation curve using 90 bp DNA and S4 IONPs. (D) Binding of target 90 bp TAMRA‐DNA onto S4 IONPs under different conditions; water, synthetic urine (SU), and physiological urine (PU).

Due to the highly cationic nature of the guanidine groups, dsDNA adsorption efficiency increased progressively from S5 to S1 (Figure S8). Specifically, S1 IONPs required approximately 66% less nanoparticle‐to‐DNA (wt/wt) ratio than S4 to achieve maximum adsorption. However, this enhanced binding affinity compromised subsequent DNA recovery, resulting in reduced elution efficiency. Consequently, the S1 formulation is better suited for applications such as sample cleanup, where strong DNA sequestration is desired, and target recovery is not required.

Particle charge plays a critical role in fragment size selectivity during electrostatically driven binding. In a mixture containing short (60 bp) and long (1500 bp) DNA, the highly cationic S1 particles demonstrated a significantly stronger initial binding bias toward 60 bp fragments compared to the less cationic S4 particles. Although S4 also favored shorter fragments, likely due to diffusion limitations and the reduced mobility of longer DNA, this bias was substantially less pronounced (Figure S9).

Bound DNA was subsequently eluted using three in‐house buffers; 1 mm EDTA in 10 mm Tris (pH 9), 1 mm Na_2_HPO_4_ in 10 mm Tris (pH 9), and 1 mm Na_2_HPO_4_ in 10 mm MES (pH 5), to evaluate the effects of pH and salt composition on DNA desorption (Figure 3B). Because the histidine moiety imparts weaker electrostatic forces, the S4 nanoparticles (80% His) achieved the highest recovery of short dsDNA fragments when using a phosphate buffer at pH 9. The linker ratio in S4 IONPs balances the high‐p*K_a_*, relatively pH‐insensitive positive charge of guanidine, which provides electrostatic anchoring and maintains a surface pI above 8 to support efficient DNA capture, with the lower‐p*K_a_*, pH‐responsive ionization of histidine, which facilitates DNA release upon changing the pH. S4 therefore represents a minimum charge scaffold that retains sufficient positive surface character for efficient DNA capture while providing the pH responsiveness required for effective release and was consequently selected for further optimization of urinary cfDNA extraction.

Polyphosphate salts enabled more efficient DNA desorption than orthophosphate salts, with a 20% increase in recovery of 30 bp dsDNA (Figure S5). This enhancement is attributed to the highly charged, polymeric structure of polyphosphates, which more effectively competes for binding sites on the IONPs and effectively displaces the adsorbed DNA. Furthermore, mild heating to 45°C, below the annealing temperature of DNA, further improved release efficiency (Figure S6). Based on these findings, an elution buffer containing 1 mm polyphosphate was selected. This formulation enabled successful DNA quantification and robust downstream amplification, yielding cycle threshold values comparable to those of DNA amplified in water (Figure S7). Although a higher concentration (10 mm) marginally improved DNA recovery, it inhibited DNA amplification, limiting its utility for downstream applications such as low‐template samples, amplicon‐based sequencing, and next‐generation sequencing (NGS) library preparation.

S4 IONPs required approximately 30 µg of IONPs per ng of dsDNA to achieve >90% adsorption (Figure 3C). Binding efficiency remained consistent in both water and synthetic urine (SU) at pH 8, representing the upper physiological range of urinary pH. Moreover, only a modest reduction (<10%) in binding efficiency was observed in human physiological urine (PU), despite the presence of competing species such as endogenous cfDNA, genomic DNA, proteins, and other urinary components (Figure 3D).

### 2.3 Nanoparticle Binding to Physiological Urinary cfDNA

The workflow for cfDNA extraction and elution from physiological urine is illustrated in Figure 4A. Although the initial IONP input (60 µg/mL of urine) was guided by spike‐in experiments in synthetic urine, its performance was subsequently validated in physiological urine, a complex matrix containing salts, cellular debris, and proteins.

**FIGURE 4:**
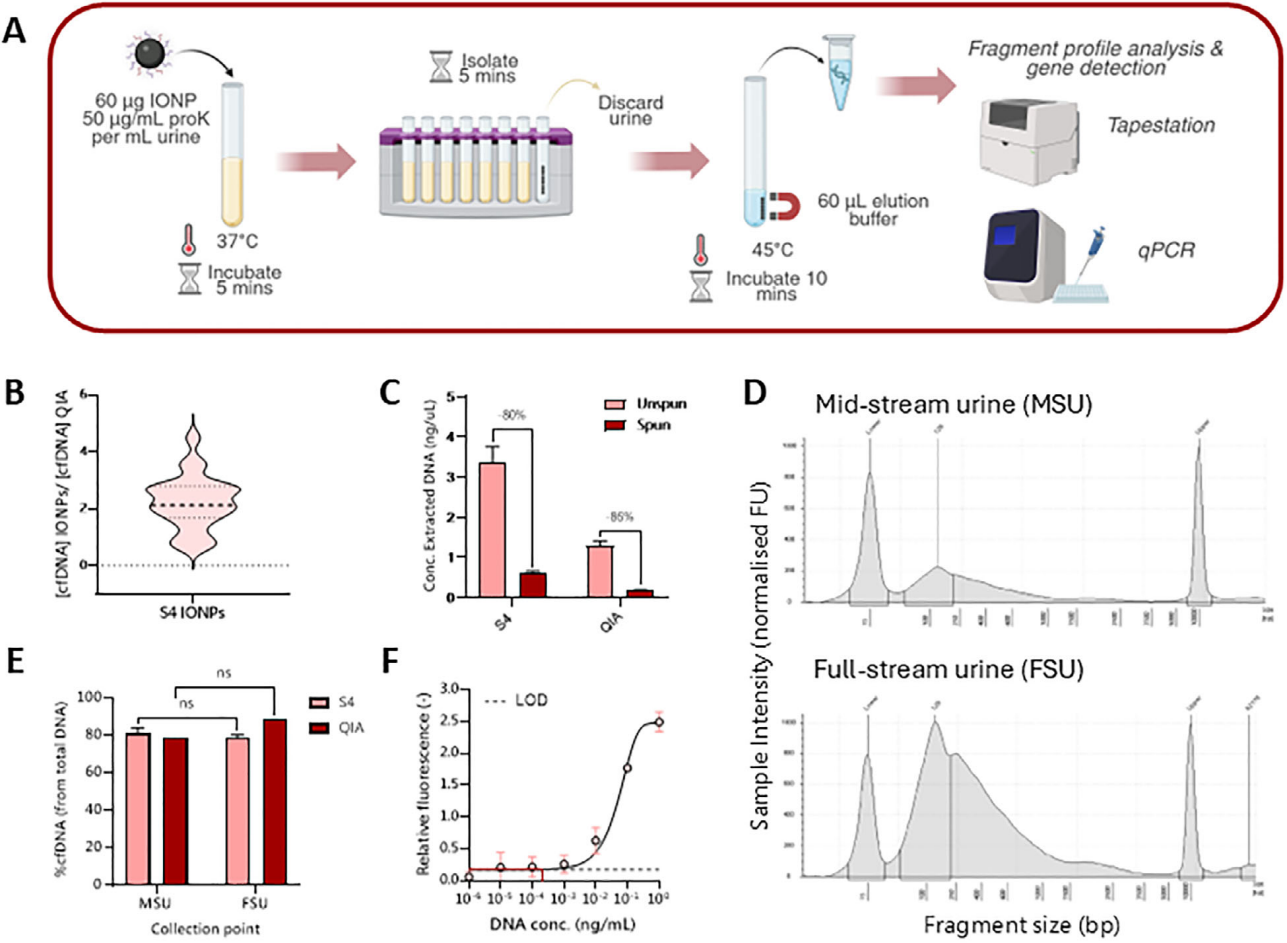
(A) Schematic representation of the experimental pipeline used for the capture and release of native urinary cfDNA. (B) Relative cfDNA yield from urine; values represent the ratio of cfDNA extracted using IONPs compared to the standard Qiagen kit from equal starting volumes (*n* = 8 biological replicates, with 3 technical repeats each). (C) Comparison of DNA extracted from spun urine versus unspun urine using nanoparticles and Qiagen kit. (D) DNA fragment profiles extracted from unspun physiological urine via mid‐stream urine collection and full‐stream urine collection using IONPs and measured using a high sensitivity D5000 tape. (E) Comparison of %cfDNA from total DNA extracted from urine in a mid‐stream vs full stream collection for both IONPs and Qiagen kit. (F) Limit of detection (LOD) of target −135 bp‐DNA in spiked physiological urine, using qPCR, where background DNA was kept constant during total DNA extractions. LOD was calculated using the Mean_blank_+3SD_blank_ based on 5 blank measurements.

To facilitate potential at‐home sampling and extraction, the protocol was adapted to rely on accessible and clinically compatible parameters: a capture temperature of 37°C, a rapid 5‐minute isolation time, and magnetic separation at 3200 Gauss, a field strength compatible with devices permitted for postal distribution to patients. The stabilized nanoparticles dispersed quickly into the urine, requiring minimal agitation, with no visible signs of aggregation during the binding incubation timeframe.

The minimum elution volume was determined to be 60 µL per mL of urine, as lower volumes resulted in a highly concentrated IONP suspension (>1 µg/µL) that impaired effective magnetic isolation. Maintaining this threshold minimized IONP carryover and ensured a clean eluate suitable for downstream analysis. Notably, Qubit fluorometry revealed a twofold increase in total DNA yield using the IONP‐based protocol compared to a standard Qiagen commercial kit for equivalent urine input volumes (Figure 4B).

Standard pre‐analytical processing for liquid urine biopsies typically involves high‐speed centrifugation (2000 x *g* for 10 min) and often the addition of EDTA to inhibit nuclease digestion prior to storage. However, to more accurately model a decentralized, at‐home capture scenario, urine samples processed with the IONP protocol were used without pre‐treatment. While centrifugation reduced the total extracted DNA yield (Figure 4C), this reduction was primarily attributable to the loss of high molecular weight (HMW) DNA (Figure S10). Although some low molecular weight (LMW) DNA associated with cellular structures or protein complexes is unavoidably lost during centrifugation, this step is traditionally employed to enrich for LMW DNA fragments. Importantly, HMW DNA (>1000 bp) retains diagnostic value for urological malignancies, such as bladder cancer [39], where localized shedding introduces tumor‐derived HMW DNA into the urine. In contrast, the diagnostic utility of trans‐renal HMW DNA for non‐urological cancers remains uncertain, as it predominantly originates from background cellular debris, including immune cells and exfoliated epithelial cells from the genitourinary tract.

Although designed for at‐home sampling, this protocol is also suitable for cfDNA extraction in clinical settings. Clinical laboratories commonly store centrifuged urine samples at −80°C without added preservatives. Under such conditions, our platform is expected to be directly applicable following sample thawing. Preservatives are sometimes used, especially when samples are transported. Therefore, an IONP‐based extraction of clinical urine samples containing Streck urine preservative at a 1:20 preservative‐to‐urine ratio was performed. DNA extraction remained successful after the samples were stored for 3 days at 4°C or room temperature (Figure S11), providing preliminary evidence that the platform can tolerate this preservation condition.

TapeStation electrophoresis of the extracted DNA revealed a primary peak between 80 and 150 bp (Figure 4D), consistent with nucleosomal (167 bp) or characteristically fragmented urinary cfDNA [40]. To evaluate potential variability in DNA profiles, extractions were performed using both mid‐stream (MSU) and full‐stream (FSU) urine collections. FSU samples yielded a higher total DNA content, with only a marginal increase in HMW DNA compared to MSU samples (Figure 4E). However, subsequent analysis using cfDNA‐specific quantification assays indicated that these differences were not significant, with comparable fragment distributions observed between MSU and FSU samples (Figures 4D and S12).

Finally, the quantitative PCR (qPCR) limit of detection for cfDNA was evaluated using a target‐sized dsDNA amplicon spiked into physiological urine. The target fragment was generated by extracting genomic DNA from EGFR‐upregulated glioblastoma cells and amplifying the sequence with EGFR‐specific primers to produce a 134 bp amplicon. The amplicon was spiked into samples at concentrations as low as 10^−6^ ng/mL of urine prior to extraction using the optimized IONP protocol.

With the IONP concentration maintained at 60 µg/mL, the system enabled simultaneous capture of endogenous urinary cfDNA and the spiked‐in target sequences. Subsequent qPCR quantification with the EGFR‐specific primers successfully detected the spike‐in DNA, establishing a lower limit of detection of 10^−3^ ng/mL. This sensitivity is comparable to that reported for plasma‐based liquid biopsy analysis [41], supporting the suitability of this approach for low‐abundance cfDNA detection (Figure 4F).

### 2.4 Mitigation of Protein‐Driven Interactions for Enhanced cfDNA Recovery

cfDNA often exists in association with proteins, forming complexes such as transiently wrapped histones or bound transcription factors, which protect it from nuclease degradation [40]. Although the overall protein content in urine is significantly lower than in blood due to glomerular filtration, the urinary proteome is dominated by proteins such as albumin and uromodulin (Tamm‐Horsfall protein) [42, 43]. While cfDNA can bind directly to proteins like albumin, uromodulin uniquely forms polymeric‐like networks [42] that can physically entrap and limit the mobility of small extracellular components, including DNA (Figure 5A).

**FIGURE 5:**
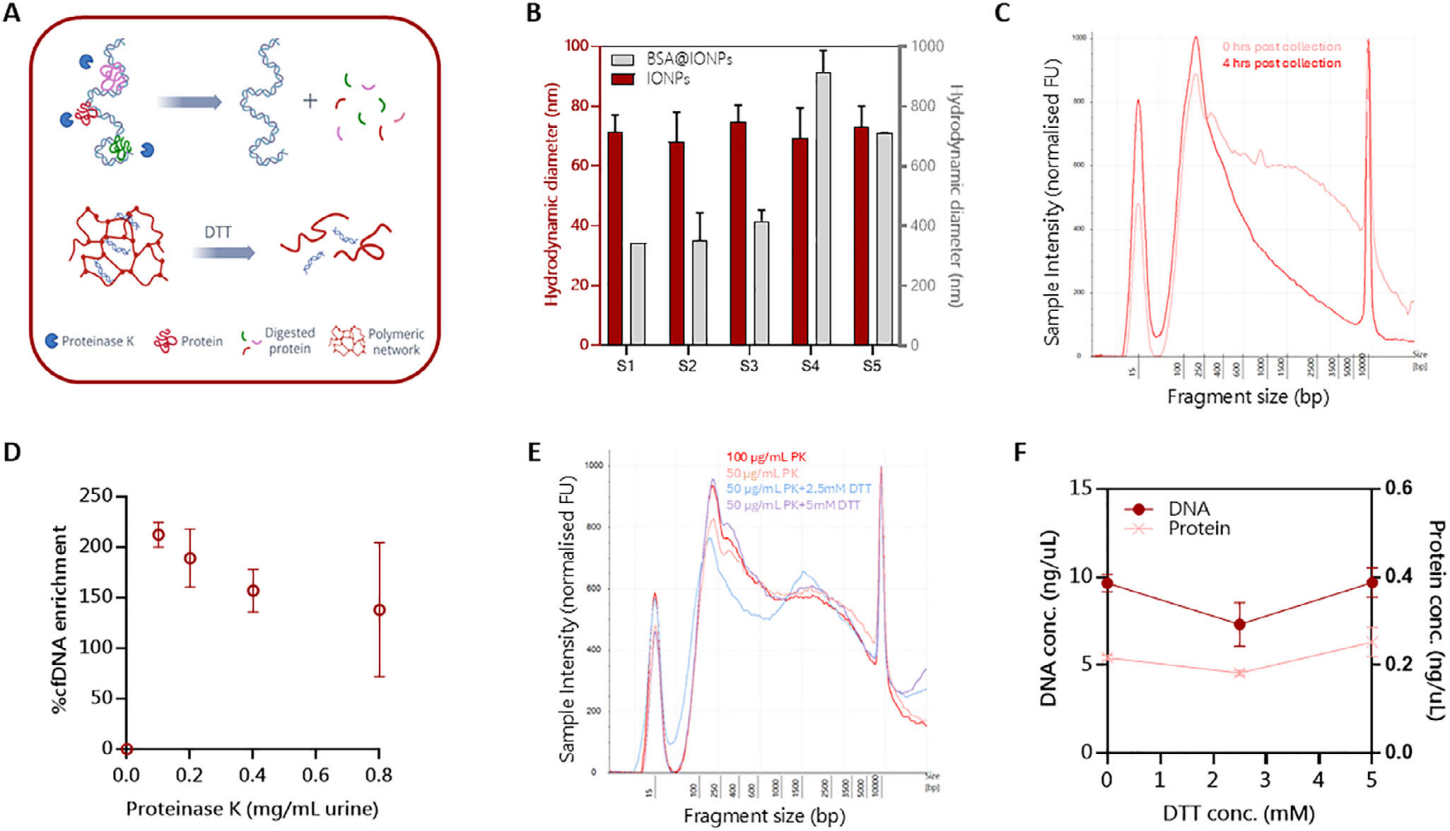
(A) Schematic representation of DNA‐protein complexes present in urine and methods for releasing protein‐bound or entrapped DNA through protein digestion or unfolding using Proteinase K (ProK) or reducing agents. (B) Hydrodynamic size of S1‐S5 IONPs pre‐and post‐incubation with excess BSA protein at 37°C for 10 min. (C) Comparative DNA fragment profile of cfDNA extracted at the time of urine collection vs 4 h post ‐collection, using a HS D5000 TapeStation. (D) %Enrichment of cfDNA from physiological urine as a function of ProK concentration. (E) Comparative DNA fragment profile of urinary cfDNA extracted with varying concentrations of ProK and DTT. (F) Concentrations of total cfDNA and protein in IONP eluates following extraction with varying amounts of ProK and DTT, quantified with Qubit HS DNA assay and HS protein assay.

To investigate protein adsorption onto the IONPs, the particles were incubated with 0.5 mg/mL bovine serum albumin (BSA). A progressive increase in hydrodynamic size was observed from system S1 to S4 (Figure 5B). This trend correlates directly with increasing histidine content, suggesting that strong histidine‐mediated protein interactions promote nanoparticle agglomeration into larger networked structures.

In contrast, despite having the highest histidine content, S5 exhibited reduced agglomeration. Such behavior is likely due to its near‐neutral surface charge at physiological pH, which diminishes the initial electrostatic interactions required to facilitate binding with negatively charged proteins.

A major challenge in urinary liquid biopsy is bench‐top nucleic acid degradation. In unprocessed urine, apparent cfDNA concentrations can increase over time as longer genomic DNA is enzymatically cleaved into cfDNA‐sized fragments (Figure 5D). By validating our extraction method using unprocessed samples, this workflow enables immediate at‐home extraction, effectively bypassing transport‐induced artifacts such as cell lysis and nuclease‐mediated fragmentation.

To release entrapped cfDNA and inhibit nucleases, proteinase K (ProK), a broad‐spectrum serine protease, is traditionally used [44]. Given urine's low protein content relative to plasma, we successfully reduced the ProK concentration from the conventional 1 mg/mL to 0.05 mg/mL without compromising DNA yield (Figure 5C). As complete protein digestion is not strictly required, chemical denaturation was explored as an alternative strategy. While the surfactant sodium dodecyl sulfate (SDS) enhanced DNA recovery, it also induced unwanted cell lysis (Figure S13), thereby introducing genomic DNA contamination. In contrast, treatment with 5 mm dithiothreitol (DTT) for 10 min successfully disrupted protein structures, yielding a cfDNA profile comparable to those obtained with higher ProK concentrations (Figure 5E). This approach provides a cost‐effective, lysis‐free alternative that is well‐suited for scalable urine processing (Figure 5F).

Urine samples were collected from multiple individuals (male and female) representing a range of urine conditions. Both protein and creatinine concentrations were quantified for each sample prior to processing (Figure S14), with creatinine used as a reference marker for urine concentration given its relatively constant excretion rate. Male and female samples fell within a comparable moderate range of protein and creatinine (protein: ∼10–50 µg/mL; creatinine: ∼2–10 mM), with the exception of one highly concentrated sample (protein: ∼125 µg/mL; creatinine: ∼23 mM), all for which the DNA extraction was successfully achieved. This indicates that the platform is broadly compatible with variations in urine concentration and protein content encountered among different samples.

### 2.5 Next Generation Sequencing (NGS) Compatibility and Genomic Profiling

To evaluate the compatibility of the S4 extraction protocol with downstream Next‐Generation Sequencing (NGS), the extracted urinary cfDNA was subjected to shallow whole‐genome sequencing. The S4 extraction yielded high‐quality sequencing libraries comparable to the gold‐standard Qiagen (QIA) kit, with over 93% of bases achieving a Q30 score (Figure 6A). Crucially, libraries prepared directly from the S4 elution buffer performed equivalently to those that underwent buffer exchange prior to library preparation (Figure 6B).

**FIGURE 6:**
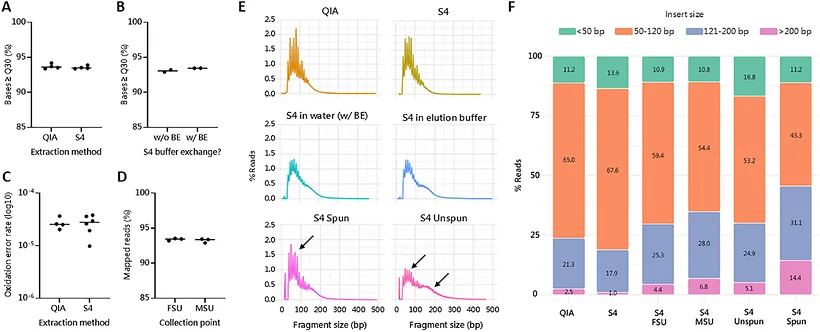
Sequencing quality metrics across extraction methods and sample types. (A‐B) Percentage of bases with quality scores ≥Q30. (C) Oxidation error rates estimated using Picard CollectOxoGMetrics (log10 scale). (D) Percentage of reads mapped to the human reference genome (hg38). Each point represents an individual library; horizontal lines indicate means. (E) Fragment size distribution profiles. (F) Relative proportion of reads categorized into four insert size bins (<50, 50–120, 121–200, and >200 bp) across extraction and pre‐analytical conditions.

This demonstrates that the custom polyphosphate elution buffer does not inhibit enzymatic library preparation steps. Furthermore, despite the potential for iron‐based nanomaterials to induce oxidative damage, the S4 protocol did not demonstrate an increased DNA oxidation error rate compared to the standard spin‐column method at this sequencing depth (Figure 6C).

In addition, mapping efficiency to the human genome remained consistently high (>93%) across both full‐stream (FSU) and mid‐stream (MSU) urine collection methods (Figure 6D). Importantly, fragment size analysis demonstrated that S4 nanoparticles efficiently capture the characteristically ultra‐short profile of urinary cfDNA. Fragment length distributions consistently exhibited a dominant peak between 50 and 120 bp across all S4 conditions (Figure 6E), in line with established urinary cfDNA signatures.

Quantitative insert size analysis further revealed the enrichment of short fragments with S4; specifically, DNA fragments <120 bp comprised 81.2% of total captured DNA, compared to 76.2% for the QIA kit (Figure 6F). While expected variations in fragment distributions were observed between spun versus unspun urine, particularly an increased proportion of larger fragments (>120 bp) in the unspun samples, the S4 system consistently demonstrated robust recovery across the full physiological size range of cfDNA. Notably, it showed a distinct advantage in capturing shorter fragments, which are often of higher diagnostic relevance.

## 3 Conclusion

In this study, we developed a rapid and highly scalable nanoparticle‐based platform for direct isolation of ultra‐low concentration cfDNA (down to 10^−3^ ng/mL) from untreated urine. By precisely tuning the surface chemistry of IONPs using guanidine and histidine linkers, we achieved optimized capture and non‐destructive release of highly fragmented DNA. A key advantage of this system is its rapid (5‐min), one‐step binding process, which eliminates the need for mechanical mixing and substantially simplifies operation. In a laboratory setting, this also eliminates long waiting times (>1 h) associated with conventional spin‐column methods for large volume urine samples, which typically require repeated loading and centrifugation or vacuum‐assisted workflows. The optimized S4 IONP protocol outperforms standard commercial silica‐based kits, yielding twofold higher total DNA recovery from equivalent sample volumes while demonstrating a comparative enrichment for the diagnostically critical short fraction (<120 bp) of urinary cfDNA.

Importantly, the platform demonstrates strong potential for volumetric scale‐up. Preliminary data demonstrates a proportional 100‐fold increase in DNA recovery when scaling the extraction from 1 to 100 mL of urine (Figure S15), highlighting its capability to seamlessly process large‐volume biological samples which is to be explored in future work. To address the complexities of the urinary matrix, a 5 mm DTT treatment was utilized to release cfDNA trapped within protein networks, entirely circumventing the cellular lysis and genomic DNA contamination characteristic of surfactant‐based extraction. Furthermore, the potential applicability of the platform to clinical workflows was addressed, demonstrating successful DNA extraction from urine stored in a Streck preservative, while noting that a comprehensive evaluation across different urine preservatives and storage conditions will be required to establish its broader clinical compatibility. Finally, the extracted DNA is fully compatible with downstream NGS applications, achieving excellent mapping efficiency (>93%) without requiring subsequent purification or buffer exchange.

By eliminating the reliance on high‐speed centrifugation, active mixing, and specialized laboratory infrastructure, this technology addresses key pre‐analytical bottlenecks in liquid biopsy workflows. Ultimately, these self‐dispersing magnetic nanoparticles represent a practical, user‐friendly platform for immediate, at‐home sample collection, enabling preservation of molecular integrity at the point of collection and advancing the clinical utility of non‐invasive cancer diagnostics and monitoring.

## 4 Experimental Section

### 4.1 Materials

All materials used in this study were purchased from Thermo Fisher Scientific, Merck, Qiagen, New England Biolabs, and Integrated DNA Technologies, and were used without further purification. All water used was Milli‐Q ultrapure grade. A detailed list of materials and suppliers is available in the Supplementary Methods.

### 4.2 Synthesis of IONPs

Fe_3_O_4_ magnetite nanoparticles were synthesized using a fed‐batch method. First, water (50 mL) was sparged with argon for 30 min to remove dissolved oxygen. The sparged water was used to prepare all the solutions needed for the synthesis. The iron precursor solution was prepared with a 2:1 ratio of Fe^2+^ to Fe^3+^. Briefly, giving a total of 0.1 m Fe concentration, FeCl_2_ (42.5 mg) and FeCl_3_·6H_2_O (180 mg) were dissolved in HCl solution (0.01 m, 10 ml) to avoid the precipitation of iron hydroxide salts.

The iron precursor solution was slowly added using a Chemyx 6000 syringe pump (0.4 mL/min) to NEt_4_OH solution (0.01 m, 20 mL), which was maintained at pH 12.0 ± 0.2. The dropwise addition ensures a homogenous reaction environment, precipitating Fe_3_O_4_ nanoparticles and allowing better control over the pH of the reaction. Finally, NEt_4_OH (0.1 m, pH 13) was prepared to control the reaction pH and was manually injected dropwise into the reaction mixture as required. Approximately 5 mL was added as the reaction pH deviated.

### 4.3 Modification of Iron Oxide Nanoparticles (S1‐S5 IONPs)

IONPs were functionalized with two different linkers: nDAG and nDAH at a molar ratio of 1:0, 5:1, 1:1, 1:5 and 0:1, respectively. The functionalization was carried out via a ‘grafting to’ method, where the linkers were initially prepared, mixed, and then slowly added onto the IONPs.

The preparation of the linkers is detailed in the Supplementary Methods. A linker solution of 2.5 mg/mL was prepared by dissolving nDAG (2.50, 1.95, 1.04, 0.31, 0.00 mg) and nDAH (0.00, 0.55, 1.46, 2.19, 2.50 mg) in 1 mL of water. The resultant solution had a pH of 5 due to traces of TFA from the synthesis of the linkers. The pH of the IONP suspension was adjusted to approximately 5 to achieve a similar acidic environment to the linker solution. The linker solution was slowly added to the IONP suspension (5 mg/mL, 2 mL), sonicated for 5 min, and mixed on a roller tube mixer for 2 h at room temperature. The modified IONPs (nDAG/H@IONPs) were washed three times using a 10 kDa PES centrifugal filter at 475 x *g* and stored at 4°C.

### 4.4 DLS and Zeta Potential Measurements

The hydrodynamic size and surface charge of the aqueous suspensions of IONPs were characterized using dynamic light scattering (DLS) and zeta potential, respectively, on a Zetasizer Nano Range instrument (Malvern Panalytical). The following specifications were used: water refractive index of 1.33, viscosity of 0.8936 mPa∙s, dielectric constant of 78.5755, and iron oxide refractive index of 2.42. Three measurements, each comprising ten sub‐runs, were taken for each sample.

### 4.5 TEM Imaging

Transmission electron microscopy (TEM) images were obtained using an FEI Tecnai F20 G2 200 kV FEGTEM equipped with a Gatan image filter (GIF) 200, followed by a 4k × 4k CCD detector to acquire the images. Samples were prepared by drop‐casting the nanoparticle suspensions (1 mg/mL, 10 µL, deionized water) onto carbon‐coated Cu grids (Agar Scientific, 400 mesh). Particle size distribution histograms were plotted by measuring at least 100 particles using the open‐source software ImageJ.

### 4.6 HPLC Analysis

To quantify the amount of nDAG and nDAH linker attached to the surface of the nanoparticles, the supernatant and washing fractions collected after synthesis were analyzed. Linker concentrations were determined by HPLC by comparing the peak area at a retention time (RT) of 9.1 min with a calibration curve (0–2 mg/mL).

For analysis, 10 µL of each sample or standard was diluted with 90 µL of methanol, and 40 µL were injected into an Agilent 1260 Infinity II HPLC system. Chromatographic separation was performed using a reverse‐phase C18 Zorbax column. The mobile phase consisted of solvent A (H_2_O containing 0.2% TFA) and solvent B (acetonitrile), delivered at a flow rate of 1 mL/min. The gradient was programmed as follows: 100% A (0–5 min), linear transition to 100% B (10 min), held at 100% B until 15 min, and returned to 100% A by 17 min. Detection was carried out at 360 nm.

### 4.7 Preparation of Fragmented DNA Samples

Genomic DNA was extracted from an EGFR‐upregulating glioblastoma cell line, U251 MG, using the Monarch Genomic DNA Purification Kit (New England Biolabs). PCR amplification of a 134 bp amplicon was done, using forward primer AGTAATGATGACTAAAGCAAGGGATTGT and reverse primer GGGATTCCGTCATATGGCTTGG, following the OneTaq 2X master mix (New England Biolabs) protocol for a 25 µL reaction. Fragment concentration was measured with a Qubit 4 Fluorometer (Invitrogen) using the broad‐range dsDNA assay, and fragment size was measured using a 4200 TapeStation system with a D1000 ScreenTape (Agilent Technologies).

### 4.8 Extraction of DNA From Synthetic Urine and Water

Synthetic fluorophore‐tagged DNA was purchased from IDT (additional information in Supplementary Methods). All experiments and associated calibration curves were performed in triplicate. First, a 90 bp DNA solution (0.1 µm, 200 µL) was prepared in synthetic urine. An optimum concentration of S4 IONP suspension (∼30 µg S4/ng DNA) was added to the DNA solution, pulse‐vortexed once, and incubated for 5 min. The suspension was then placed on a magnetic rack for 10 min to fully isolate the IONPs until the solution appeared clear.

The supernatant was pipetted into a new tube, and the remaining (unbound) DNA was quantified using a CLARIOstar Plus plate reader (BMG LABTECH) with 547 nm excitation and 589 nm emission wavelengths.

The adsorption capacity of magnetic nanoparticles was calculated as follows:

$$\%\;adsorption=\left(\frac{C_{0}-C_{r}}{C_{0}}\right)\;\times \frac{V_{0}}{V_{2}}\times 100\%$$

where *C*_0_ is the initial DNA concentration and *C_r_* is the concentration of remaining DNA in solution after the adsorption experiment. *V*_0_ is the starting DNA solution volume and *V*_2_ is the final DNA solution volume after addition of IONP suspension (*V*_0_ + *V_IONPs_*).

DNA was eluted from the S4 IONPs by adding the elution buffer (EB) (200 µL) to the IONPs. The suspension was vortexed briefly to disperse the nanoparticles and incubated for 10 min at 45°C. The elution buffer was prepared by dissolving sodium polyphosphate (4.83 mg) and Tris‐HCl (18.2 mg) in deionized water (15 mL), with the pH adjusted to 9 by the addition of NaOH solution (1 m).

The suspension was then placed on a magnetic rack for 10 min or until the solution appeared clear. The supernatant was pipetted out, and the eluted DNA was quantified with the fluorometer as described above. The percentage of DNA recovery was calculated as follows:

$$\%\;recovery=\left(\frac{C_{0}-C_{e}}{C_{0}}\right)\;\times \frac{V_{EB}}{V_{0}}\;\times 100\%$$

where *C*_0_ is the initial DNA concentration and *C_e_* is the concentration of eluted DNA in solution, *V*_0_ is the starting volume of DNA solution and *V_EB_* is the volume of elution buffer used.

### 4.9 Preparation of Urine Samples

Urine samples were collected from 8 healthy subjects. Written consent forms were obtained. To simulate home‐use conditions, DNA was isolated within an hour after sample collection without any pre‐treatment. Conversely, to remove cellular debris and reflect standard clinical pre‐processing, a subset of the samples was centrifuged prior to DNA isolation (2000 x *g*, 10 min). Samples not used immediately were stored at −80°C.

### 4.10 Extraction of Urinary cfDNA Using our Nanoparticles

S4 IONPs (60 µg) and proteinase K enzyme (50 µg) were added to fresh untreated human urine (1 mL). The suspension was incubated for 5 min and left on the magnetic rack for 5 min for full isolation of IONPs until solution appeared clear. The clear urine was pipetted out and discarded. cfDNA was eluted from S4 IONPs by the addition of the polyphosphate elution buffer (60 µL). The resulting suspension was vortexed once to disperse the nanoparticles then incubated for 10 min at 45°C. The mixture was placed on a magnetic rack for 10 min or until the solution is clear. The supernatant was pipetted out and eluted cfDNA was quantified using the HS dsDNA Qubit assay and cfDNA fragmentation profiles were analyzed using 4200 TapeStation instrument and HSD5000 tapes or cfDNA tapes.

### 4.11 Extraction of Urinary cfDNA Using the QIAamp MinElute ccfDNA Kit

cfDNA isolation was performed following the manufacturer's protocol. The reagents in the QIAamp MinElute ccfDNA Kit (Qiagen, Germany) were used to isolate cfDNA from urine samples (1 mL), which was then eluted in deionized water (60 µL).

### 4.12 Quantification of DNA by Qubit and TapeStation

The Invitrogen Qubit 4 Fluorometer was used for quantifying cfDNA concentrations, utilizing the Qubit dsDNA High Sensitivity Assay Kit purchased from Thermo Fisher Scientific. DNA fragment profiles were analyzed using the 4200 Agilent TapeStation system with High Sensitivity DNA ScreenTapes purchased from Agilent Technologies.

### 4.13 Quantification of DNA by qPCR

Quantitative PCR (qPCR) was performed using purified cfDNA obtained from healthy volunteer physiological urine. The qPCR reaction was performed on Applied Biosystems QuantStudio 5 Systems (Thermo Fisher Scientific) for a 20–100 µL total input volume. A final reaction volume of 100 µL was prepared using PowerUp SYBR Green master mix (50 µL) (Thermo Fisher Scientific), forward and reverse primers (500 nmol/L), and cfDNA eluate (50 µL) from the IONP and Qiagen kit extractions. The reaction mixture was prepared in the wells of a 96‐well PCR plate (Thermo Fisher Scientific). Finally, the plate was sealed using an Adhesive PCR plate sealer (Thermo Fisher Scientific). The standard curve was prepared using our in‐house elution buffer. Based on a reaction volume increase optimization experiment, the final thermal cycling conditions were as follows: UDG activation at 50°C for 2 min, initial enzyme activation at 95°C for 2 min, followed by 40 cycles of denaturation at 95°C for 15 s, annealing at 56°C for 15 s, and extension at 72°C for 1 min. Data were acquired and interpreted using amplification plots, and analyzed using StepOne Software (Applied Biosystems, Thermo Fisher Scientific). To calculate amplification efficiency, Ct values were plotted on a logarithmic scale against the corresponding concentrations of DNA input. Next, a linear regression curve through the data points was generated, and the slope of the trend line was calculated. Finally, efficiency was calculated using the following equation: $E\;=10^{-\frac{1}{\mathrm{slope}}}-1.$

### 4.14 Whole Genome Sequencing (WGS) of Urinary cfDNA

To validate the genomic integrity and downstream utility of the extracted urinary cfDNA, whole genome sequencing (WGS) was performed. Sequencing libraries were constructed using the ThruPLEX DNA‐Seq Kit (Takara Bio) according to the manufacturer's protocol, which is specifically optimized for low‐input and highly fragmented nucleic acids. Following library preparation and standard quality control, the multiplexed libraries were loaded onto an Illumina MiSeq i100 sequencer. Sequencing was executed using a 150 bp paired‐end (PE150) configuration, yielding a total of approximately 5 million reads distributed across the samples on the flow cell.

## 5 Data Processing

Paired‐end shallow whole‐genome sequencing data were processed using Bash scripting on a SLURM cluster. Sample identities were matched from the sequencing run sheet to the corresponding paired FASTQ files.

Raw reads were trimmed with fastp to remove adapters and low‐quality bases. Read quality was then checked with FastQC and summary measures such as Q30, read retention, duplication rate, and read length were collected from the fastp output.

Trimmed reads were aligned to the human reference genome (hg38) using bwa mem. BAM files were then processed with samtools, including sorting, mate fixing, duplicate marking, and indexing. Mapping statistics were obtained with samtools flagstat and samtools stats.

Read groups were added with Picard AddOrReplaceReadGroups. Oxidative damage was assessed with Picard CollectOxoGMetrics, and additional sequencing artifact and GC bias metrics were generated with Picard CollectSequencingArtifactMetrics and CollectGcBiasMetrics.

Fragment size information was taken from the aligned BAM files using properly paired, non‐duplicate reads. Insert sizes were summarized both as full fragment length distributions and as proportions of reads within selected size bins.

## 6 Statistical Analysis

Statistical analyses were performed using GraphPad Prism version 11.0.0. Depending on the experimental design, data were analyzed using an ordinary one‐way or two‐way analysis of variance (ANOVA), followed by Tukey's post‐hoc test for multiple comparisons. A *p*‐value < 0.05 was considered statistically significant. All data are presented as the mean ± SEM (*n* = 3 independent experiments per group, unless otherwise stated).

## Nomenclature

cfDNA: Cell‐free DNA
ctDNA: Circulating‐tumor DNA
DLS: Dynamic light scattering
EGFR: Epidermal growth factor receptor
FSU: Fill‐stream urine
FU: Fluorescence units
HPLC: High‐performance liquid chromatography
IONP: Iron oxide nanoparticles
LOD: Limit of detection
MSU: Mid‐stream urine
nDAG: Nitrodopamine‐guanidine
nDAH: Nitrodopamine‐histidine
NGS: Next generation sequencing
pI: Isoelectric point
PU: Physiological urine
QIA: Qiagen kit
qPCR: Quantitative polymerase chain reaction
SD: Standard deviation
SU: Synthetic urine
TEM: Transmission electron microscopy
VSM: Vibrating sample magnetometer

## Funding

Dina Haddad was funded by W. D. Armstrong Studentship and Cambridge Trust. Ljiljana Fruk and Ishtiaq Ahmed would like to acknowledge funding from EPSRC IRC Programme grant (EP/S009000/1) and CRUK Early Detection Primer Award (EDDPMA‐May23/100051)

## Conflicts of Interest

The authors declare no conflicts of interest.

## Supporting information




**Supporting File**: smtd71034‐sup‐0001‐SuppMat.docx.

## Data Availability

All data supporting the findings of the study are available with the paper, in electronic Supplementary Material, or from the corresponding authors upon request.
