# Supplementary material for: Nature‐Inspired Histidine and Guanidine Modification of Magnetic Nanoparticles for Urinary DNA Liquid Biopsy
Source: Small Methods. 2026 Sep 18;10(20):e71034. doi: 10.1002/smtd.71034 (PMC13706641; doi:10.1002/smtd.71034)
Supplement: Supplementary file 1 [file SMTD-10-e71034-s001.docx]

Supporting Information

**Nature-Inspired Histidine and Guanidine Modification of Magnetic Nanoparticles for Urinary DNA Liquid Biopsy**

Dina Haddad, Ishtiaq Ahmed, Manthos Pitoulias, Richard Mair, Ljiljana Fruk*

** Corresponding author*

D. Haddad (dsyh2@cam.ac.uk) - CEB/CRUK, I. Ahmed, L. Fruk ([lf389@cam.ac.uk](mailto:lf389@cam.ac.uk)) - CEB, M. Pitoulias, R. Mair - CRUK

Chemical Engineering and Biotechnology (CEB), University of Cambridge, Cambridge, United Kingdom

Cancer Research UK Cambridge Institute (CRUK), University of Cambridge, Cambridge, United Kingdom

**Table S1** Main techniques for DNA isolation and purification including examples of commercial kits and key specifications.

| **Technique** | **Kit and manufacturer name** | **Input volume**  **(mL)** | **Elution volume**  **(µL)** | **Minimum size (bp)** | **Compatible biofluids** | | **Source** |
| --- | --- | --- | --- | --- | --- | --- | --- |
| **Silica spin-column** | QIAamp ccfDNA Kit^a^  **(Qiagen)** | 1-5 | 20-150 | 20 | Plasma, serum, urine^b^ | ^[1]^ | |
|  | Quick-cfDNA Serum & Plasma Kit  **(Zymo)** | 1-10 | ≥35 | 100 |  | ^[2]^ | |
|  | NucleoSnap cfDNA kit  **(Macherey-Nagel)** | 1–10 | 20-100 | 50 |  | ^[3]^ | |
|  | Norgen Plasma/Serum Cell-Free DNA Maxi Kit  **(Norgen)** | 5-10 | 50-100 | 50 |  | ^[4]^ | |
|  | Quick-DNA Urine Kit  **(Zymo)** | ≤40 | ≥ 10 | 100 | Urine | ^[5]^ | |
|  | Norgen Urine Cell-Free Circulating DNA Maxi Kit  **(Norgen)** | 10-30 | 50-100 | 50 | Urine | ^[6]^ | |
| **Magnetic bead-based** | MagMAX™ Cell-Free DNA Isolation Kit **(Thermo Fisher)** | 0.5-10 | 15–50 | 80 | Plasma, serum, urine^b^ | ^[7,8]^ | |
|  | NextPrep-Mag URINE cfDNA Isolation Kit **(Bioo Scientific / PerkinElmer)** | 4-20 | 30-100 (manual)  75-100 (automated) | 100 | Urine | ^[9]^  based on Cat#3826-02 on manufacturer website | |
| **Organic extraction/ precipitation**  (lab use only) | Phenol-chloroform followed by isopropanol/ethanol precipitation | User-defined | User-defined | N/A | Plasma, serum, urine | ^[10,11]^ | |
| ^a^ Combination of magnetic beads and silica spin column  ^b^ Listed as compatible by manufacturer, though kit primarily intended and validated for other biofluids | | | | | | | |

**Figure S1.** Schematic of the fed-batch reactor setup used for the coprecipitation synthesis of iron oxide nanoparticles.

**Figure S2.** Comparative cost breakdown of raw materials required to synthesize 1 g of iron oxide nanoparticles (IONPs) using coprecipitation versus microemulsion methods.

**Figure S3.** Zeta potential of bare IONPs as a function of pH. Nanoparticles were dispersed in water and pH was adjusted with NaOH or HCl.

**Figure S4**. **A** Vibrating sample magnetometer (VSM) measurement of 7 nm IONPs synthesised with coprecipitation and 13 nm IONPs synthesised with microemulsion. **B** Transmission electron microscopy image and size distribution histogram of the 13 nm IONPs.

**Figure S5.** Percentage 30 bp dsDNA recovered from S4 IONPs using elution buffer with Na_2_HPO_4_ vs Na polyphosphate salts, both buffered to pH 9.

**Figure S6.** Effect of heating (to 45 °C) and shaking (600 rpm) during the elution step on the percentage of DNA recovered.

**Figure S7. A** Amplification plot and cycle threshold values of urinary cfDNA, using EGFR primers. cfDNA was extracted using Qiagen Kit and eluted in water (blue), diluted to a final 10 mM and 1 mM polyphosphate solution (dark purple and light purple) and 10 mM Tris buffer (green). **B** Efficiency of the amplification in 1 mM polyphosphate buffer, using three 10-fold dilutions of cfDNA.

**Figure S8.** Percentage DNA adsorbed onto IONPs (S1-S5) using sheared dsDNA with a modal peak at 150 bp in synthetic urine.

**Figure S9. Differential adsorption of 60 bp and 1500 bp DNA fragments by S1 and S4 IONPs.** **A** Electrophoretic profiles of the starting DNA mixture and the unbound DNA remaining in the supernatant after adding varying amounts of S1 or S4 IONPs (prepared at equal stock concentrations). **B** Adsorption efficiency (%) of the 60 bp and 1500 bp fragments from the mixture using both IONP systems, plotted across a nanoparticle-to-DNA mass ratio of 0-0.8 µg/ng.

**Figure S10. Impact of pre-extraction centrifugation on urinary cfDNA profiles.** TapeStation profiles of cfDNA from two biological samples (left and right) comparing uncentrifuged and centrifuged urine. Centrifugation leads to a depletion of high-molecular-weight (HMW) DNA (resulting in apparent LMW DNA enrichment) and a reduction in total DNA yield, including the LMW fraction.

**Figure S11.** **Effect of Streck urine preservative on the cfDNA recovery using IONPs.** TapeStation profiles of cfDNA extracted from a urine sample on day 1 without a preservative, and the same urine sample stored at 4°C and room temperature for 3 days with a 1:20 preservative-to-urine ratio.

**Figure S12.** **cfDNA recovery from full-stream (FSU) vs. mid-stream urine (MSU).** **A** TapeStation profiles comparing the percentage of target cfDNA (50-700 bp) extracted from FSU and MSU using S4 IONPs. **B** Comparison of total DNA yields obtained from FSU and MSU using S4 IONPs versus the Qiagen kit.

**Figure S13. Impact of SDS on cfDNA extraction with S4 IONPs.** TapeStation profiles displaying cfDNA recovery across varying SDS concentrations, highlighting the SDS threshold at which cell lysis occurs, introducing high-molecular-weight genomic DNA contamination into the sample.

**Figure S14. Creatinine and protein concentrations of untreated urine from 15 healthy individuals.** Creatinine and protein assays were performed on unspun and untreated urine prior to cfDNA extraction.

**Figure S15. Scalability and specificity of large-volume cfDNA extraction. A** 3D CAD design of a high-volume urine bath (250 mL capacity and 25 mm width) compatible with a standard DynaMag (commercial) magnet, used for magnetic nanoparticle recovery from large urine volumes. The vessel was 3D printed using what PLA basic filament on a Bambu P1S printer. **B** Comparison of DNA and protein yields recovered from 1 mL and 100 mL urine samples. Processing 100 mL of urine resulted in a 100-fold increase in DNA yield, while non-specific protein co-isolation increased by only 8-fold.

**Materials and methods**

*Materials*

| **Material** | **Catalogue number** | **Supplier** |
| --- | --- | --- |
| Di-tert-butyl decarbonate | 205249 | Merck |
| 2,2′-(ethylenedioxy)bis(ethylamine) | 385506 |  |
| Dopamine hydrochloride | H8502 |  |
| N,N-Diisopropylethylamine | D125806 |  |
| Boc-His-OH | 408433 |  |
| Triethylamine | 8083520100 |  |
| N, N’-Di-Boc-1H-pyrazole-1-carboxamidine | 434167 |  |
| Boc-GABA-OH | 469572 |  |
| 2-(3,4-dihydroxyphenyl)acetic acid | [ENA372572979](https://www.sigmaaldrich.com/GB/en/product/enamine/ENA372572979?context=bbe) |  |
| Dichloromethane | [270997](https://www.sigmaaldrich.com/GB/en/product/sial/270997) |  |
| Tetrahydrofuran | [34865](https://www.sigmaaldrich.com/GB/en/product/sigald/34865) |  |
| Trifluoroacetic acid | [T6508](https://www.sigmaaldrich.com/GB/en/product/sigald/T6508) |  |
| HATU | [445460](https://www.sigmaaldrich.com/GB/en/product/aldrich/445460) |  |
| Magnesium sulfate | M2643 |  |
| Sodium sulfate | [239313](https://www.sigmaaldrich.com/GB/en/product/sigald/239313) |  |
| Sodium nitrate | 239313 |  |
| Sulfuric acid | 258105 |  |
| Ethyl acetate | [319902](https://www.sigmaaldrich.com/GB/en/product/sigald/319902) |  |
| EDTA | [E9884](https://www.sigmaaldrich.com/GB/en/product/sial/E9884) |  |
| Hydrochloric acid | [258148](https://www.sigmaaldrich.com/GB/en/product/sigald/258148) |  |
| Sodium polyphosphate | 1.06529 |  |
| Sodium phosphate dibasic | [S9763](https://www.sigmaaldrich.com/GB/en/product/sigald/S9763) |  |
| Tris hydrochloride | [10812846001](https://www.sigmaaldrich.com/GB/en/product/roche/10812846001) |  |
| [Iron(III) chloride hexahydrate](https://www.sigmaaldrich.com/GB/en/product/aldrich/940534) | [940534](https://www.sigmaaldrich.com/GB/en/product/aldrich/940534) |  |
| [Iron(II) chloride](https://www.sigmaaldrich.com/GB/en/product/aldrich/372870) | [372870](https://www.sigmaaldrich.com/GB/en/product/aldrich/372870) |  |
| [Tetraethylammonium hydroxide](https://www.sigmaaldrich.com/GB/en/product/mm/822149) | [8.22149](https://www.sigmaaldrich.com/GB/en/product/mm/822149) |  |
| Dithiothreitol | 10197777001 |  |
| Sodium dodecyl sulfate | 436143 |  |
| Dimethylamine | 426458 |  |
| Acetonitrile | [34851](https://www.sigmaaldrich.com/GB/en/product/sigald/34851) |  |
| Methanol | [34860](https://www.sigmaaldrich.com/GB/en/product/sigald/34860) |  |
| Human genomic DNA | 69237-M |  |
| Nitrodopamine hydrosulfate | WAA58149 | Biosynth |
| High Sensitivity D5000 ScreenTape | 5067-5592 | Agilent |
| High Sensitivity D5000 Reagents | 5067-5593 |  |
| Cell-free DNA ScreenTape | 5067-5630 |  |
| Cell-free DNA Reagents | 5067-5631 |  |
| PowerUp™ SYBR™ Green Master Mix | A25742 | Thermo Fischer Scientific |
| Qubit High Sensitivity DNA assay | Q32854 |  |
| Qubit Protein assay | Q33212 |  |
| One*Taq^®^* 2X Master Mix with Standard Buffer | M0482S | New England Biolabs |
| ThruPLEX® DNA-Seq Kit | R400674 | Takara Bio |

**Fluorophore-labelled dsDNA**

HPLC-purified dsDNA oligos: ATTO(488)-labelled 25 bp and TAMRA-labelled 90 bp were purchased from Integrated DNA Technologies (IDT) and dissolved in 10 mM Tris, 0.1 mM EDTA. Different fragment lengths comprised repeating units of the same sequence. DNA are the following:

30 bp sense sequence: /5ATTO488N/CGA ACC CTG CCC AGG TCG ACA CAT AGG TGA

90 bp sense sequence: /55-TAMK/CGA ACC CTG CCC AGG TCG ACA CAT AGG TGA GGT CTG CTA CCG AAC CCT GCC CAG GTC GAC ACA TAG GTG AGG TCT GCT ACC GAA CCC TGC

**Nitrodopamine-guanidine linker (IAC-242) synthesis**

**1. Nitrodopamine (IAC-211)**

Nitrodopamine (**IAC-211**) was synthesized according to the literature with slight modification.^[12]^ 20% sulphuric acid (5 ml) was cautiously and very slowly added to a cooled solution of dopamine hydrochloride (1 g, 5.29 mmol) and NaNO_2_ (1.26 g, 23.8 mmol) in water (35 ml) under vigorous stirring. The yellow solid which immediately precipitated was collected by suction filtration and washed several times with cold water and cold methanol. (1.25 g, 4.22 mmol, 80%).

**^1^H NMR (500 MHz, DMSO-*d_6_*)**: δ (ppm) 3.06 (bs, 4H), 5.40 (br, 1H), 6.85 (s, 1H), 7.46 (s, 1H).

**HR-MS (ESI):** [M+H]^+^ Calculated for C_8_H_11_N2O_4_: 199.09651; found 199.09692

***NMR spectra for Nitrodopamine (IAC-211)***

**2. Protected nitrodopamine guanidine (IAC-241)**

**IAC-241** was prepared according to the literature with slight modification.^[13]^ Nitrodopamine (**IAC-211**) (1.0 g, 5.01 mmol, 1 equiv.) was dissolved in anhydrous DMF (15 mL) and triethylamine (1.53 g, 2.05 mL, 15.03 mmol, 3 equiv.) was added. The solution was cooled to 0 ^o^C and N, N'-Di-Boc-1*H*-pyrazole-1-carboxamidine (1.86 g, 6.01 mmol, 1.2 equiv) was then added portion wise. The resulting mixture was allowed to warm to room temperature and stirred overnight. The solvent was evaporated under reduced pressure. The residue obtained was dissolved in dichloromethane (50 mL) and washed with water (20 mL) and brine (20 mL). The organic phase was dried over anhydrous MgSO_4_, filtered and evaporated under reduced pressure to afford crude product which was purified by flash silica gel chromatography (1:1 Hex:EtOAc) to produce (**IAC-241**) as yellowish solid (1.82 g, 4.15 mmol, 83% yield).

**^1^H NMR (500 MHz, DMSO-*d_6_*)**: δ (ppm) 1.41 (s, 9H), 1.45 (s, 9H), 3.01 (t, *J* = 6.7 Hz, 2H), 3.54 (q, *J* = 5.7 Hz 2H), 6.69 (s, 1H), 7.51 (s, 1H), 8.28 (s, 1H), 7.62 (s, 1H), 8.49 (m, 1H), 10.14 (br, 1H), 11.43 (bs, 1H).

**^13^C NMR (125 MHz, DMSO-*d_6_*)**: δ (ppm) 27.6, 27.7, 28.0, 32.0, 40.5, 78.1, 81.9, 82.9, 112.3, 118.3, 127.6, 139.6, 144.0, 151.1, 151.3, 151.9, 155.3, 163.1.

**HR-MS (ESI):** [M+H]^+^ Calculated for C_19_H_29_N_4_O_8_: 441.19071; found 441.19580

***NMR spectra for protected nitrodopamine guanidine (IAC-241)***

**3. Nitrodopamine guanidine (IAC-242)**

To a solution of nitrodopamine boc-protected guanidine (**IAC-241**) (1.0 g, 2.27 mmol) in dichloromethane (15 mL) was added trifluoroacetic acid (10 mL). The mixture was stirred for 3 hours at room temperature. The solvent was evaporated to give light yellow residue. Dichloromethane (20 mL) was added to the residue and evaporated. This process was repeated three times (3x20 mL) to remove the trifluoroacetic acid. Toluene (30 mL) was added to the light brown residue and the solvent was evaporated in order to remove the traces of trifluoroacetic acid. Crude product was purified by flash chromatography (20% MeOH in DCM) to give a yellow solid of nitrodopamine guanidine (**IAC-242**) (501 mg, 2.09 mmol, 92% yield).

**^1^H NMR (500 MHz, DMSO-*d_6_*)**: δ (ppm) 2.98 (t, *J* = 6.7 Hz, 2H), 3.34 (q, *J* = 7.0 Hz, 2H), 6.78 (s, 1H), 7.54 (s, 1H), 7.67, (t, *J* = 5.7 Hz, 1H), 10.04 (br, 1H), 10.44 (br, 1H).

**^13^C NMR (125 MHz, DMSO-*d_6_*)**: δ (ppm) 32.2, 41.2, 112.4, 118.5, 126.9, 139.4, 144.3, 151.5, 156.9.

**HR-MS (ESI):** [M+H]^+^ Calculated for C_9_H_13_N_4_O_4_: 241.09389; found 241.09362

***NMR spectra for nitrodopamine guanidine (IAC-242)***

**Nitrodopamine-histidine linker (IAC-283) synthesis**

**1. tert-butyl (R)-(1-((4,5-dihydroxy-2-nitrophenethyl)amino)-3-(1H-imidazol-4-yl)-1-oxopropan-2-yl)carbamate (IAC-282)**

To a stirred solution of Boc-His-OH (1.0 g, 3.92 mmol) and HATU (1.61 g, 4.24 mmol) in 30 mL of anhydrous DMF was added DIPEA (1.36 g, 1.78 mL, 10.60 mmol). The reaction mixture was cooled to 0 ^o^C and a solution of nitrodopamine **IAC-211** (0.93 g, 4.7 mmol) in 10 mL anhydrous DMF was added dropwise over a period of 10 min. The reaction mixture was stirred at room temperature overnight. The solvent was removed and the residue was dissolved in 100 mL EtOAc and washed with water (3×50 mL), brine (50 mL) and dried over anhydrous sodium sulfate. The combined organic layer was concentrated to give yellow oil which was purified by silica gel chromatography eluting with CH_2_Cl_2_ to CH_2_Cl_2_/MeOH (10:1) to afford boc-protected compound **IAC-282** as pale-yellow solid (1.38 g, 3.18 mmol, 82% yield).

**^1^H NMR (400 MHz, CD_3_OD):** δ (ppm) 1.36 (s, 9H). 2.85-3.01 (m, 3H), 3.14-3.19 (m, 1H), 3.42-3.50 (m, 2H), 4.32-4.41 (m, 1H), 6.71 (br, 1H), 7.26 (br, 1H), 7.50 (br, 1H), 8.71 (br, 1H).

**^13^C NMR (100 MHz, CD_3_OD):** δ (ppm) 28.2, 30.4, 33.5, 40.7, 54.6, 58.0, 80.8, 113.0, 118.0, 118.8, 131.0, 134.5, 141.3, 144.9, 151.9, 157.1, 172.4.

**HR-MS (ESI):** [M+H]^+^ calculated for C_19_H_26_N_5_O_7_ [M+H]+: 436.1827 found 436.1819

***tert-butyl (R)-(1-((4,5-dihydroxy-2-nitrophenethyl)amino)-3-(1H-imidazol-4-yl)-1-oxopropan-2-yl)carbamate (IAC-282)***

**2. (R)-2-amino-N-(4,5-dihydroxy-2-nitrophenethyl)-3-(1H-imidazol-4-yl)propenamide (IAC-283)**

Trifluoroacetic acid (8 mL) was added to a solution of boc-protected **IAC-282** (500 mg, 1.15 mmol) in 15 mL dry CH_2_Cl_2_. The reaction mixture was stirred at room temperature for 4 h. CH_2_Cl_2_ (20 mL) was then added and the solvent was removed under reduced pressure. The procedure was repeated three times to remove traces of TFA to afford the title compound (**IAC-283**) as brown oil in quantitative yield.

**^1^H NMR (400 MHz, CD_3_OD):** δ (ppm) 2.91-2.96 (m, 2H), 3.02-3.12 (m, 1H), 3.18-3.26 (m, 1H), 3.40-3.50 (m, 1H), 3.54-3.61 (m, 1H), 3.63-3.73 (m, 1H), 4.18 (m, 1H), 6.75 (br, 1H), 7.52 (br, 1H), 8.42 (br, 1H), 8.71 (br, 1H).

**^13^C NMR (100 MHz, CD_3_OD):** δ (ppm) 32.6, 39.6, 51.9, 54.4, 112.1, 117.9, 120.8, 128.4, 128.4, 140.1, 144.0, 150.7, 167.1

**HR-MS (ESI):** [M+H]^+^ calculated for C_14_H_18_N_5_O_5_ [M+H]+: 336.1302 found 336.1294

***NMR spectra of (R)-2-amino-N-(4,5-dihydroxy-2-nitrophenethyl)-3-(1H-imidazol-4-yl)propenamide (IAC-283)***

**References**

[1]	QIAamp MinElute ccfDNA Kits: Plasma & Serum cfDNA | QIAGEN, .

[2]	Quick-cfDNA Serum & Plasma Kit | ZYMO RESEARCH – Zymo Research International, .

[3]	NucleoSnap cfDNA kit for cell-free DNA from plasma, MN | MACHEREY-NAGEL, .

[4]	Plasma/Serum Cell-Free Circulating DNA Purification Kits | Norgen Biotek Corp., .

[5]	Quick-DNA Urine Kit – Zymo Research International, .

[6]	Urine Cell-Free Circulating DNA Purification Kits | Norgen Biotek Corp., .

[7]	MagMAX^TM^ Cell-Free DNA Isolation Kit 1 Kit | Buy Online | Applied Biosystems^TM^, .

[8]	B. M. M. Wever, R. D. M. Steenbergen, *Mol. Oncol.* **2024**, *19*, 1918.

[9]	G. Streleckiene, H. M. Reid, N. Arnold, D. Bauerschlag, M. Forster, *Biotechniques* **2018**, *64*, 225.

[10]	X. Xue, M. D. Teare, I. Holen, Y. M. Zhu, P. J. Woll, *Clin. Chim. Acta* **2009**, *404*, 100.

[11]	H. Yuan, Z. Z. Zhu, Y. Lu, F. Liu, W. Zhang, G. Huang, G. Zhu, B. Jiang, *Yonsei Med. J.* **2012**, *53*, 132.

[12]	A. Napolitano, M. d’Ischia, C. Costantini, G. Prota, *Tetrahedron* **1992**, *48*, 8515.

[13]	A. Y. Zhao, A. F. Brooks, D. M. Raffel, J. Stauff, J. Arteaga, P. J. H. Scott, X. Shao, *ACS Med. Chem. Lett.* **2020**, *11*, 2325.
